# Mitochondrial Genome Architecture of *Cephus nigrinus* Thomson, 1871 and the Conserved IQM Rearrangement in Cephinae (Hymenoptera: Cephidae)

**DOI:** 10.3390/biology15212009

**Published:** 2026-11-05

**Authors:** Sevda Hastaoğlu Örgen

**Affiliations:** Department of Biology, Faculty of Science, Sivas Cumhuriyet University, 58140 Sivas, Türkiye; hastaoglu@cumhuriyet.edu.tr

**Keywords:** *Cephus nigrinus*, Cephidae, Cephinae, mitochondrial genome architecture, gene rearrangement, comparative mitogenomics, molecular evolution, phylogeny, Symphyta

## Abstract

Mitochondrial gene rearrangements provide useful characters for investigating genome evolution in Hymenoptera, but mitogenomic sampling within Cephidae remains limited. Here, the mitochondrial genome of *Cephus nigrinus* Thomson, 1871 was reconstructed from publicly available Illumina whole-genome sequencing data (SRA: SRR23907852) and compared with available cephid mitochondrial genomes. The assembled sequence is 16,782 bp long, has an A + T content of 81.38%, and contains 13 protein-coding genes, 22 transfer RNA genes, and two ribosomal RNA genes. An A + T-rich region of 1295 bp was identified in the deposited annotation. Comparative gene-order analysis revealed a conserved rearrangement of the ancestral mitochondrial IQM cluster, characterized by the positioning of trnQ before trnI near *nad2* and the relocation of trnM immediately downstream of rrnS. Phylogenetic analyses based on a concatenated dataset of 13 protein-coding genes, 22 tRNAs, and two rRNAs recovered *C. nigrinus* and *C. pygmeus* as sister taxa with strong support. However, the placement of this pair relative to *C. cinctus* received lower support in the maximum-likelihood analysis. The results expand available mitogenomic information for Cephinae and support conservation of the rearranged mitochondrial gene organization among the examined *Cephus* species. Further validation is needed to establish the complete structure of the repetitive control region.

## Simple Summary

Stem sawflies (Cephidae) are a relatively understudied group of Hymenoptera, and mitochondrial genome data remain limited for several species. *Cephus nigrinus* is a Palearctic sawfly associated mainly with wild grasses. In this study, we reconstructed its mitochondrial genome using publicly available Illumina sequencing reads and compared its gene organization with those of related species. The 16,782-bp sequence contains the typical set of 37 mitochondrial genes and exhibits a conserved rearrangement involving the positions of three transfer RNA genes. The annotated A + T-rich region is relatively short compared with that of some congeners, although its complete structure remains to be independently verified. Phylogenetic analyses recovered *C. nigrinus* as the sister taxon of *C. pygmeus*. These findings provide additional information about mitochondrial genome organization and evolutionary relationships within Cephidae.

## 1. Introduction

Mitochondrial genomes have long served as valuable molecular resources for investigating evolutionary relationships and species boundaries in insects. Their relatively compact organization, predominantly maternal inheritance, conserved orthologous gene content, and generally elevated substitution rates make them suitable for molecular systematics, species identification, population genetics, and phylogeographic studies [1,2]. Compared with individual mitochondrial markers such as cox1, complete mitogenomes provide a considerably larger number of phylogenetically informative characters, including nucleotide composition, codon usage, evolutionary rates, RNA secondary structures, and gene-order rearrangements. Consequently, comparative mitogenomic analyses can improve phylogenetic resolution, clarify uncertain taxonomic relationships, and facilitate the discrimination of closely related or morphologically similar insect species [2,3,4].

In insects, mitogenomes are typically double-stranded circular molecules ranging from approximately 14 to 25 kb in length, although substantially longer genomes occur in several lineages because of expansion of the control region. The canonical mitogenome comprises 37 genes—13 protein-coding genes (PCGs), 22 transfer RNA genes (tRNAs), and two ribosomal RNA genes (rRNAs)—together with a major non-coding control region, commonly referred to as the A + T-rich region [5,6,7,8]. The protein-coding genes encode essential components of the oxidative phosphorylation system, whereas the two rRNA genes and 22 tRNA genes contribute to mitochondrial protein synthesis. The control region contains regulatory elements involved in the initiation of mitochondrial DNA replication and transcription and frequently accounts for variation in mitogenome length because of its highly variable nucleotide composition, tandem repeats, and other repetitive motifs [6,7]. Although mitochondrial gene content is generally conserved among insects, gene order can vary considerably across lineages, particularly through translocation, inversion, duplication, or loss of tRNA genes. Such rearrangements may represent lineage-specific or shared derived characters and therefore provide additional evidence for reconstructing phylogenetic relationships and clarifying the evolutionary history of insect groups [6,7,8,9].

The rapid development of next-generation sequencing (NGS) technologies, together with the growing availability of publicly accessible raw sequencing data in repositories such as the NCBI Sequence Read Archive (SRA), has greatly accelerated the recovery of complete and nearly complete insect mitogenomes. Mitogenomes can be reconstructed from high-throughput sequencing datasets, including sequencing libraries generated for broader genomic investigations, thereby expanding the availability of mitochondrial genomic resources [10]. Despite these advances, mitogenomic representation remains uneven across hymenopteran lineages. This imbalance is especially pronounced among early-diverging lineages traditionally grouped within the paraphyletic grade Symphyta, in which taxon sampling is still incomplete at both the family and genus levels [11,12,13,14,15].

Comparative analyses have shown that tRNA rearrangements are not restricted to the derived suborder Apocrita. In Symphyta, rearrangements are concentrated mainly in the trnI–trnQ–trnM (IQM) and trnW–trnC–trnY (WCY) clusters, and occasionally involve trnL1 or additional tRNA blocks [11,13,15]. Relative to Apocrita, symphytan protein-coding genes generally evolve more slowly and remain under purifying selection, yet lineage-specific architectural features are nonetheless phylogenetically informative [11,15]. Mitogenomic and nuclear phylogenomic studies consistently recover Symphyta as a paraphyletic grade at the base of Hymenoptera and place Cephoidea close to the origin of parasitoid and apocritan lineages, typically as sister to Orussoidea + Apocrita after the radiation of eusymphytan superfamilies [12,13,14,16]. Increased sampling within Cephoidea is therefore relevant not only to family-level systematics but also to the reconstruction of early hymenopteran genome evolution.

**Figure 1:**
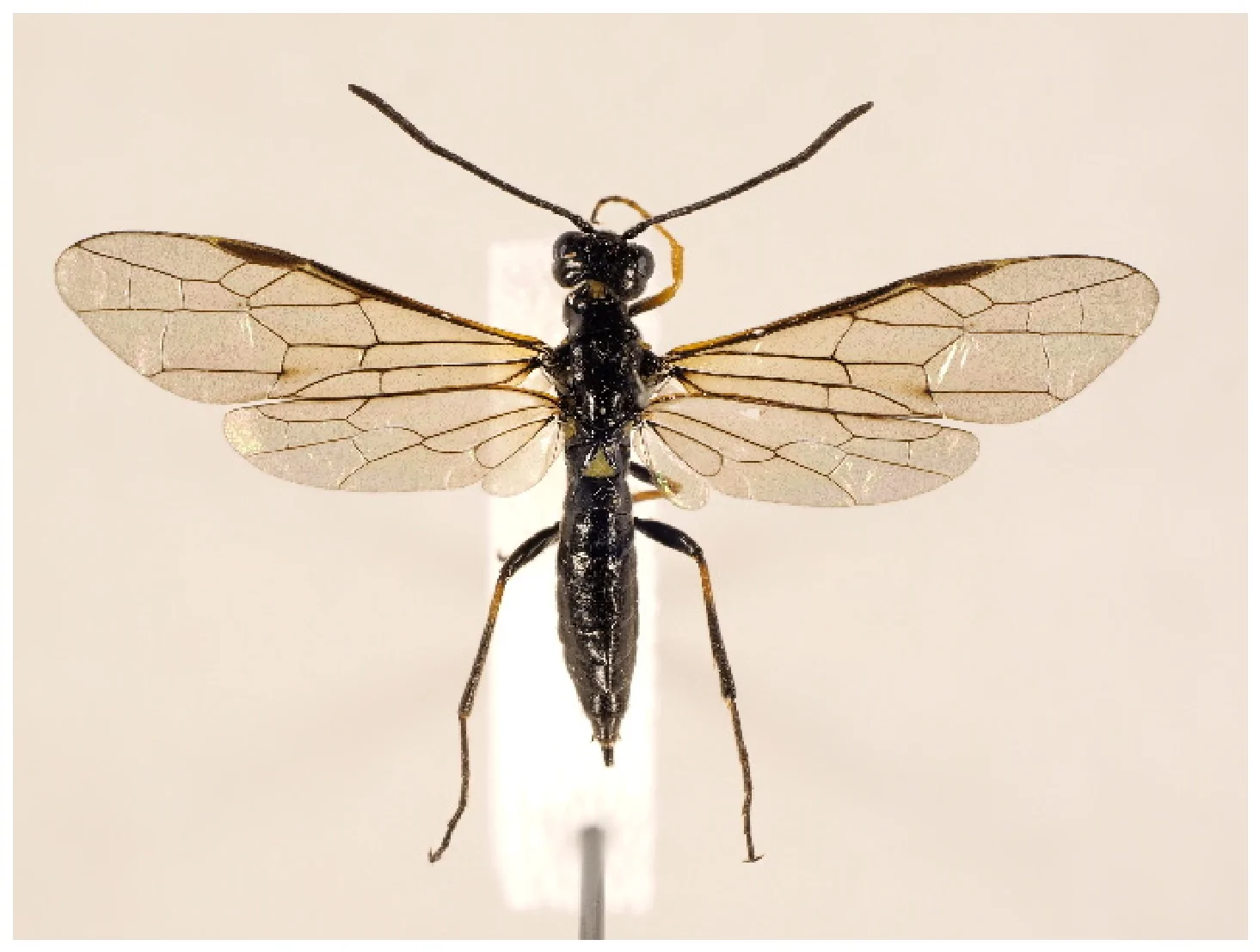
Adult specimen of *Cephus nigrinus*. Photograph by Marko Mutanen (University of Oulu), sourced from BOLD Systems (Sample ID: ZMUO.032754), licensed under Creative Commons Attribution Non-Commercial 4.0 International (2018) [26].

The family Cephidae (stem sawflies) remains comparatively underrepresented relative to its morphological and ecological diversity. Species of the genus *Cephus* are distributed throughout the Palearctic and Nearctic regions. Several species, notably *C. cinctus*, *C. pygmeus* and related Cephini, are of agricultural importance because their endophytic larvae develop within the stems of grasses and cereal crops, weakening culms, promoting lodging and reducing yield [16,17,18]. Other *Cephus* species, including *C. nigrinus* Thomson, 1871, have also been documented in regional faunistic studies, contributing to our understanding of the distribution and diversity of Cephidae [19]. An adult specimen of *C. nigrinus* is shown in Figure 1. Expanding mitogenomic resources for both pest and non-pest cephids is important for improving phylogenetic resolution within Symphyta and for investigating mitochondrial gene arrangement, genome organization, codon usage and nucleotide composition across Hymenoptera [17,18,20].

The first complete cephid mitogenome, that of the Nearctic wheat stem sawfly *C. cinctus*, already revealed derived tRNA positions relative to the ancestral insect gene order [9,21]. Subsequent work on Palearctic Cephini demonstrated a suite of shared mitochondrial characters, including rearrangement of the IQM cluster, an unusual elongation of rrnS not reported elsewhere in Hymenoptera, and extensive tandem-repeat expansion of the A + T-rich region [17,18,22]. Broader sampling across the subfamily Cephinae further showed that IQM rearrangement is a synapomorphy of the subfamily, that rearrangements also affect the trnT–trnP cluster, and that tRNA duplication events otherwise rare in arthropods—and previously unknown in other Symphyta—occur in this lineage [20]. These studies likewise indicated that the traditional tribal classification of Cephinae (Cephini, Hartigiini and Pachycephini) is not fully recovered by mitochondrial or combined mitochondrial–nuclear datasets, and that diversification of the subfamily is closely associated with host-plant colonization [20,23]. Previous studies have also reported parallel amino acid substitutions in mitochondrial protein-coding genes of Cephini, although the evolutionary mechanisms underlying these substitutions require further investigation [18]. Mitogenomic information remains limited for several lineages of Cephidae, particularly outside Cephini. The mitochondrial genome of *Pachycephus smyrnensis* has contributed additional information on mitochondrial genome organization outside Cephini [24]. Within *Cephus*, available mitochondrial genome records include *C. cinctus*, *C. pygmeus*, *C. sareptanus*, and *C. spinipes* [9,17,25]. However, some deposited sequences are incomplete, particularly in their terminal and non-coding regions, limiting direct comparisons of total genome length and control-region structure. The mitochondrial genome of *C. nigrinus* provides an additional dataset for investigating conserved gene arrangements and mitochondrial sequence variation within the genus.

In the present study, we reconstructed and annotated the mitochondrial genome of *Cephus nigrinus* Thomson, 1871 using publicly available Illumina whole-genome sequencing reads deposited in the NCBI Sequence Read Archive (SRA accession: SRR23907852). We characterized its mitochondrial gene content, nucleotide composition, codon usage, and gene organization, with particular emphasis on the IQM-associated rearrangement previously reported in Cephinae. We further compared the mitochondrial gene arrangements of *C. nigrinus*, *C. cinctus*, *C. pygmeus*, and *C. sareptanus* using their available GenBank annotations. Phylogenetic relationships were investigated using a concatenated dataset comprising 13 protein-coding genes, 22 tRNAs, and two rRNAs. By integrating mitochondrial genome annotation, comparative gene organization, and phylogenetic analyses, this study contributes additional evidence for understanding mitochondrial genome evolution within Cephidae.

## 2. Materials and Methods

### 2.1. Mitogenome Assembly and Coverage Analysis

The publicly available whole-genome sequencing dataset of *Cephus nigrinus* was retrieved from the NCBI Sequence Read Archive under accession SRR23907852 (BioProject PRJNA809169; BioSample SAMN28561985). The dataset was generated using the Illumina HiSeq X Five platform (Illumina, Inc., San Diego, CA, USA) with 150-bp paired-end sequencing and comprised approximately 69.3 million read pairs. The present study used these previously deposited data for mitochondrial genome reconstruction; specimen collection, DNA extraction, and primary sequencing were not performed as part of this analysis.

Prior to assembly, raw sequencing reads were quality- and adapter-trimmed using Trimmomatic v0.39 [27]. Reference-guided mapping of the filtered reads was performed using BBMap v38.90 [28] (maxindel = 20, mappedonly = t), with the mitochondrial genome of *Cephus pygmeus* (GenBank accession KM377623) as the reference. This analysis was conducted to identify mitochondrial reads within the whole-genome sequencing dataset and assess their sequence similarity to the reference mitochondrial genome. The final *C. nigrinus* mitochondrial genome was reconstructed independently through de novo assembly using SPAdes (https://github.com/ablab/spades; accessed on 23 October 2026) [29], as detailed in Section 2.2, and deposited in GenBank under accession BK071385.

**Figure 2:**
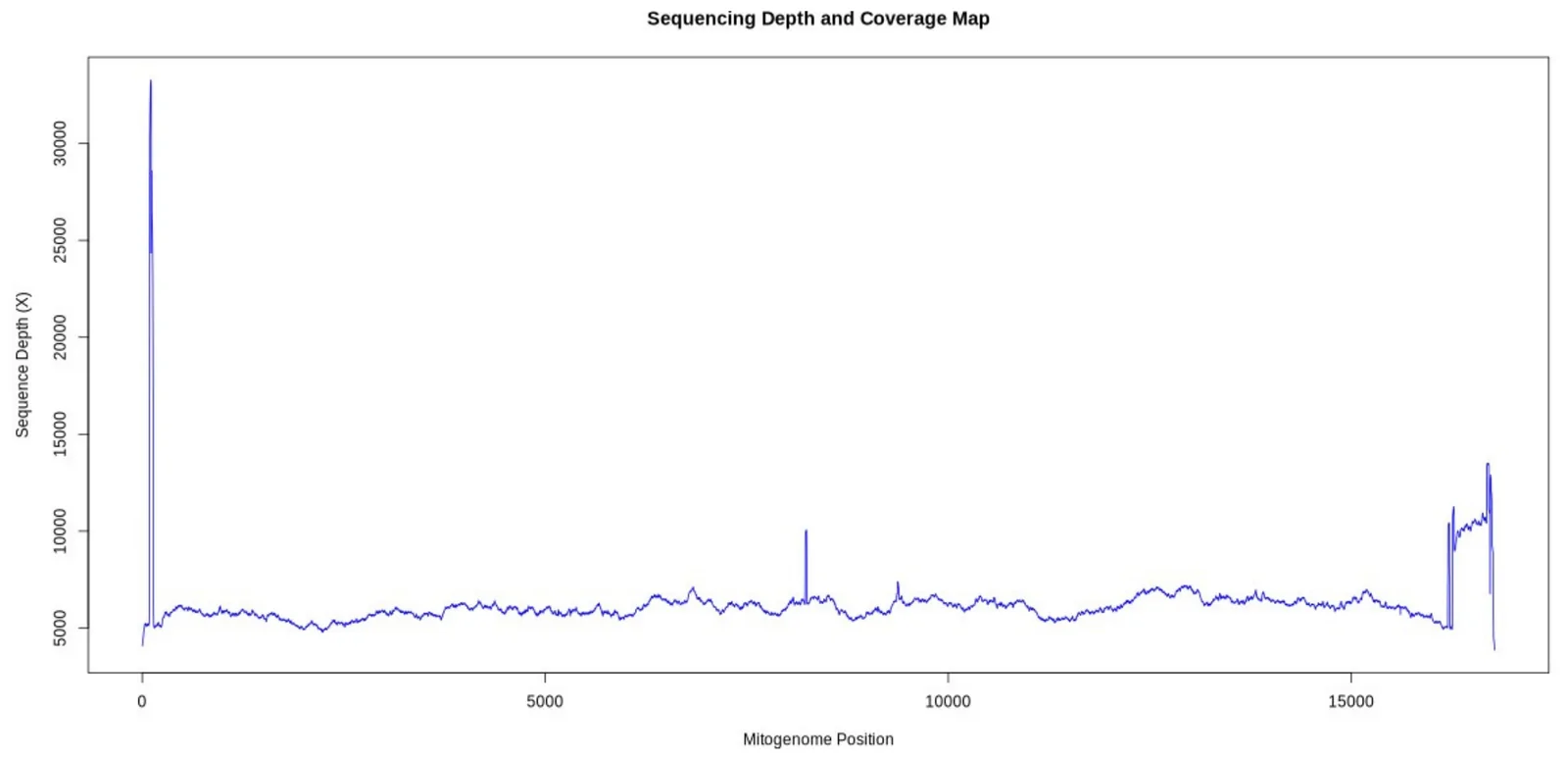
Sequencing depth across the 16,782-bp mitochondrial genome assembly of *Cephus nigrinus*. Original paired-end Illumina reads were mapped to the de novo assembled mitochondrial sequence using BWA, and per-base coverage was calculated using SAMtools. The x-axis represents nucleotide position (bp), and the y-axis represents sequencing depth (×).

For post-assembly coverage assessment, Trimmomatic-filtered paired-end reads were mapped to the 16,782-bp de novo assembled *C. nigrinus* mitochondrial sequence using BWA-MEM v0.7.17 [30] with default alignment parameters. Properly paired alignments with a minimum mapping quality (MAPQ) score of 30 were retained using SAMtools (https://www.htslib.org/; accessed on 23 October 2026) [31] (samtools view -f 2 -q 30). Per-base sequencing depth was calculated using SAMtools depth -a, and the resulting coverage profile was visualized using the ggplot2 package (https://ggplot2.tidyverse.org/; accessed on 23 October 2026) in R (https://www.r-project.org/; accessed on 23 October 2026) [32] (Figure 2). A minimum sequencing depth of 10× was used as the criterion for evaluating base-level coverage. The assembled mitochondrial sequence exhibited a mean coverage depth of 6219.26×, a median depth of 6047.5×, and a minimum depth of 3864×. All 16,782 nucleotide positions exceeded the specified coverage threshold, and the deposited sequence contained no ambiguous nucleotide positions (N). The mitochondrial genome deposited under accession BK071385 was derived from de novo assembly rather than reference-guided consensus reconstruction; post-assembly mapping was performed to assess sequencing coverage and did not contribute to the generation of the deposited sequence.

### 2.2. De Novo Assembly and Mitogenome Annotation

Quality filtering of the Illumina reads was performed using Trimmomatic [27], followed by de novo assembly with SPAdes [29]. Candidate mitochondrial contigs were identified through BLAST similarity searches (https://blast.ncbi.nlm.nih.gov/Blast.cgi; accessed on 23 October 2026) against available hymenopteran mitochondrial sequences. The mitochondrial sequence obtained through de novo assembly was selected for downstream annotation and comparative analyses. Mitochondrial gene annotation was performed using the MITOS2 web server (https://mitos.bioinf.uni-leipzig.de/; accessed on 23 October 2026) [33] with the invertebrate mitochondrial genetic code (NCBI genetic code 5). The predicted gene boundaries and coding sequences were subsequently inspected in Geneious Prime (https://www.geneious.com/; accessed on 23 October 2026). The annotation was examined for the presence and arrangement of the 13 protein-coding genes (PCGs), 22 transfer RNA genes (tRNAs), and two ribosomal RNA genes (rRNAs). Predicted tRNA secondary structures were obtained using MITOS2. The final annotated mitochondrial sequence was deposited in GenBank under accession BK071385.

For comparative gene-order analysis, annotated mitochondrial genome records of *C. cinctus* (FJ478173), *C. nigrinus* (BK071385), *C. pygmeus* (KM377623), and *C. sareptanus* (KM377624) were retrieved from GenBank. The order and strand orientation of protein-coding, tRNA, and rRNA genes were compared using the annotated feature coordinates. The gene arrangements were aligned at *nad2* for visualization, and the comparative diagram was generated using Python (https://www.python.org/; accessed on 23 October 2026) and Matplotlib (https://matplotlib.org/; accessed on 23 October 2026).Missing regions in partial GenBank records were not interpreted as gene losses.

Nucleotide composition and strand asymmetry were assessed using AT-skew = (A − T)/(A + T) and GC-skew = (G − C)/(G + C) [34]. These indices were calculated for the mitochondrial genome and relevant gene categories. Relative synonymous codon usage (RSCU) was evaluated for the mitochondrial protein-coding genes using MEGA X (V10) [35].

The presence of the expected mitochondrial gene complement and the absence of ambiguous nucleotides were assessed in the final assembly. However, circularization of the mitochondrial molecule and the complete repeat structure of the A + T-rich region were not independently confirmed. Therefore, the assembled sequence is described according to the available sequence and annotation evidence without claiming experimentally verified circularity or complete resolution of the control region.

### 2.3. Phylogenetic Analyses

To investigate the phylogenetic position of *Cephus nigrinus*, mitochondrial sequences representing 17 taxa, including the outgroup *Cladiucha punctata*, were analyzed. The final dataset comprised 13 protein-coding genes (PCGs), 22 transfer RNA genes (tRNAs), and two ribosomal RNA genes (rRNAs). *Cladiucha punctata* (Tenthredinidae; NC_062838) was selected as an outgroup because it belongs to a hymenopteran family outside Cephidae, providing an external reference for rooting the ingroup phylogeny.

Protein-coding genes were aligned using an amino acid-guided approach in MEGA, applying the invertebrate mitochondrial genetic code (NCBI genetic code 5). The corresponding nucleotide sequences were retained for phylogenetic inference. Transfer RNA and ribosomal RNA sequences were aligned using MAFFT v7 [36]. The individual gene alignments were concatenated using SequenceMatrix v1.7.8 [37], resulting in a nucleotide dataset comprising 16,619 aligned positions for 17 taxa. Terminal stop codons were excluded from the PCG alignment.

PartitionFinder v2 [38] was used to evaluate partitioning strategies based on mitochondrial gene regions and codon positions. The selected scheme comprised six subsets, five assigned GTR + I + G and one assigned HKY + I + G. PartitionFinder results were examined separately from the substitution-model selection performed for the maximum-likelihood analysis. The detailed partitioning scheme, including the assigned mitochondrial gene regions, codon positions, and selected substitution models, is provided in Supplementary Table S2.

Bayesian phylogenetic inference (BI) was performed using MrBayes v3.2.7 [39], applying the six-partition scheme identified by PartitionFinder. Partition-specific substitution models were implemented accordingly: the GTR + I + G model was assigned to the first five subsets using ‘lset applyto = (1,2,3,4,5) nst = 6 rates = invgamma’, while the HKY + I + G model was specified for the sixth subset using ‘lset applyto = (6) nst = 2 rates = invgamma’. Substitution-rate parameters (‘revmat’), state frequencies (‘statefreq’), gamma shape parameters (‘shape’), and proportions of invariant sites (‘pinvar’) were unlinked across partitions using ‘unlink statefreq = (all) revmat = (all) shape = (all) pinvar = (all)’. Branch lengths remained linked across partitions, whereas relative evolutionary rates were allowed to vary using ‘prset applyto = (all) ratepr = variable’. Default MrBayes prior distributions were retained, including Dirichlet priors for substitution rates and nucleotide frequencies, a uniform prior for tree topology, and an exponential prior for branch lengths. Two independent Markov chain Monte Carlo (MCMC) runs (‘nruns = 2’), each comprising four Metropolis-coupled chains (one cold and three heated; ‘nchains = 4’), were performed for 5,000,000 generations, with sampling every 1000 generations. The initial 25% of sampled trees (‘burninfrac = 0.25’) were discarded as burn-in, and the remaining samples were used to construct a 50% majority-rule consensus tree. MCMC convergence was assessed using the average standard deviation of split frequencies (ASDSF < 0.01) and potential scale reduction factors (PSRF approaching 1.000) obtained from MrBayes, together with effective sample sizes (ESS > 200) evaluated using Tracer v1.7 [40].

Maximum-likelihood (ML) phylogenetic inference was conducted using IQ-TREE v1.6.12 [41]. ModelFinder selected the GTR + F + I + G4 model [42] under the Bayesian information criterion (BIC). Branch support was evaluated using 1000 ultrafast bootstrap [43] replicates and the SH-aLRT test [44].

The ML and BI consensus trees were compared to assess agreement in the inferred relationships, particularly within *Cephus*. Branch support is reported separately as ultrafast bootstrap percentages for ML and posterior probabilities for BI. To further evaluate potential sources of phylogenetic uncertainty, exploratory substitution saturation and nucleotide-composition heterogeneity analyses were performed on the mitochondrial protein-coding gene dataset. First, the three codon positions were examined separately by comparing pairwise nucleotide differences, including transitions and transversions, against patristic distances obtained from the original ML phylogeny. Paired-sequence symmetry tests were also conducted to assess deviations from compositional homogeneity and substitutional symmetry [45]. In addition, a supplementary maximum-likelihood analysis was performed using only the first and second PCG codon positions to investigate the sensitivity of the inferred relationships to the exclusion of rapidly evolving third-position sites. The results of these supplementary analyses are presented in Supplementary Figure S1 (exploratory substitution saturation), Supplementary Table S1 (paired-sequence symmetry tests), and Supplementary Figure S2 (ML analysis based on the first and second PCG codon positions). The resulting ML and BI phylogenetic trees were visualized using FigTree v1.4.2 [46]. The taxonomic identities and GenBank accession numbers of all taxa included in the phylogenetic analyses are summarized in Table 1.

## 3. Results

The assembled mitochondrial sequence of *Cephus nigrinus* was 16,782 bp long and contained the typical complement of 37 mitochondrial genes, comprising 13 protein-coding genes (PCGs), 22 transfer RNA genes (tRNAs), and two ribosomal RNA genes (rRNAs) (Table 2). Based on the deposited GenBank annotation (BK071385), 23 genes were located on the positive (+) strand and 14 on the negative (−) strand. The sequence also included an annotated A + T-rich region of 1295 bp.

The mitochondrial gene arrangement differed from the putative ancestral insect organization, particularly in the positions of the tRNA genes associated with the ancestral IQM cluster. In C. nigrinus, trnQ preceded trnI near nad2, whereas trnM was located immediately downstream of rrnS and upstream of the A + T-rich region (Table 2). The mitochondrial sequence exhibited a pronounced A + T bias, with an overall A + T content of 81.38%. The highest A + T content among PCG codon positions was observed at the third position (90.15%). The combined PCGs showed an A + T content of 78.53%. Nucleotide composition and AT- and GC-skew values for the different mitochondrial gene categories are summarized in Table 3.

**Figure 3:**
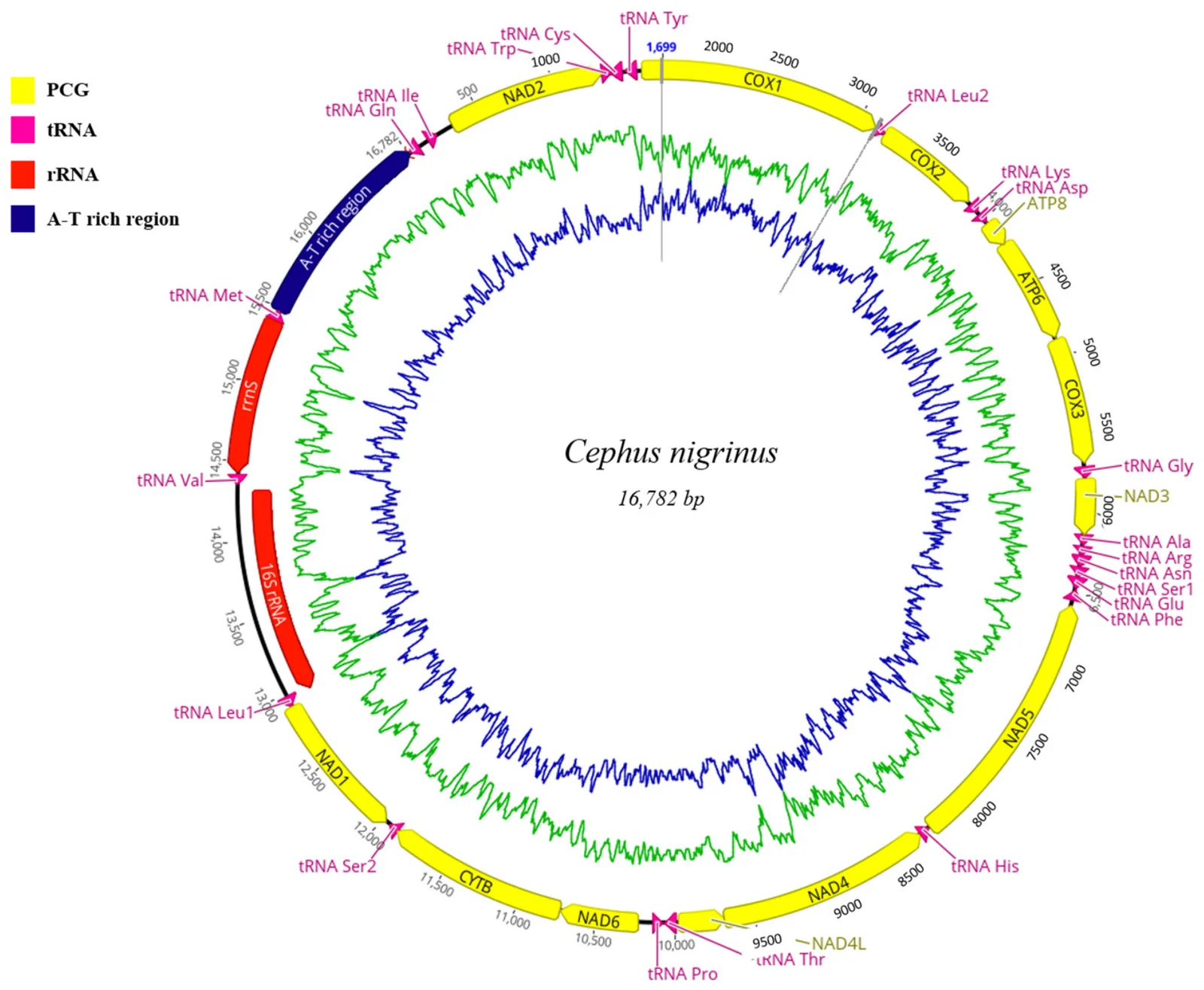
Mitochondrial genome map of *Cephus nigrinus* (BK071385), generated using Geneious Prime. The outer circle illustrates the positions and orientations of the annotated protein-coding genes (PCGs), transfer RNA genes (tRNAs), ribosomal RNA genes (rRNAs), and the A + T-rich region. The inner green and blue plots represent local G + C and A + T nucleotide contents, respectively. The circular layout is a graphical representation of the annotated sequence and does not independently establish genome circularity.

Read mapping to the de novo assembled mitochondrial sequence showed a mean sequencing depth of 6219.26× and a median depth of 6047.5×, with coverage ranging from 3864× to 33,269× (Figure 2). All 16,782 nucleotide positions were covered by mapped reads, and the final assembled sequence contained no ambiguous nucleotides. Elevated coverage peaks were observed near the terminal regions of the linearized assembly, particularly in the vicinity of the annotated A + T-rich region. These peaks may reflect repetitive sequences, uneven read mapping, or sequence-composition effects; however, their precise origin could not be established from coverage depth alone. The mitochondrial genome organization of *C. nigrinus* is illustrated in Figure 3.

**Figure 4:**
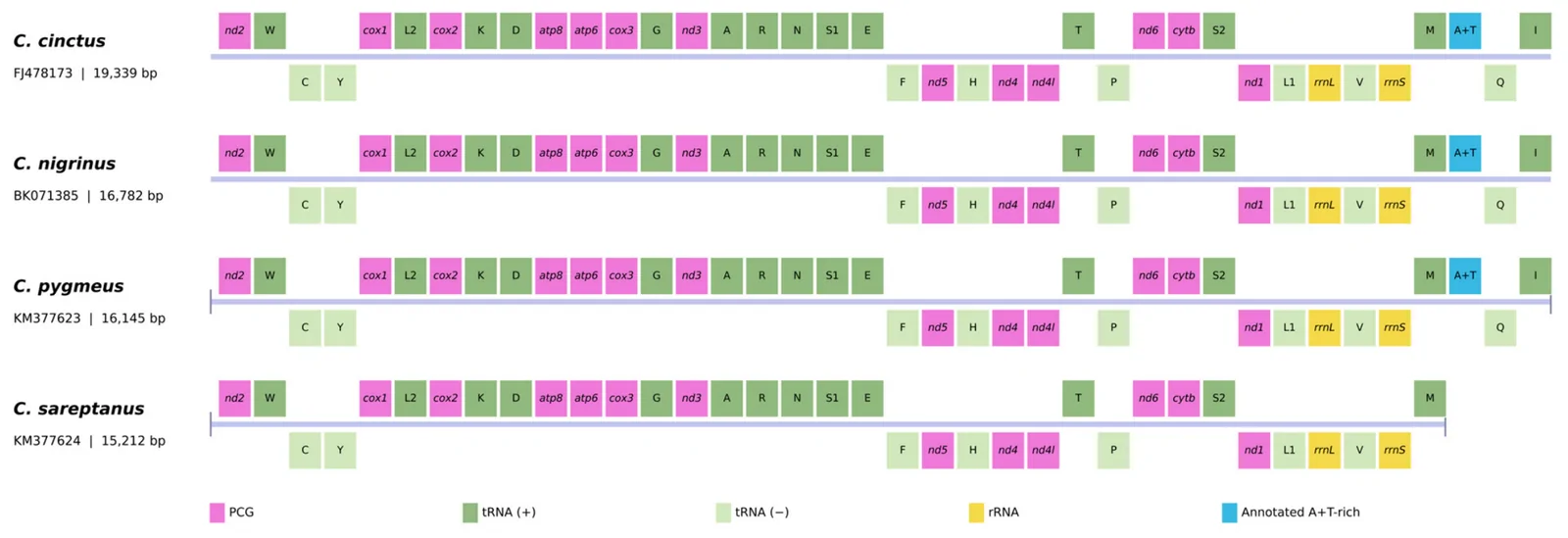
Comparative mitochondrial gene organization in four *Cephus* species. Mitochondrial gene arrangements of *C. cinctus* (FJ478173), *C. nigrinus* (BK071385), *C. pygmeus* (KM377623), and *C. sareptanus* (KM377624) were compared using their GenBank annotations. Gene arrangements were aligned at *nad2* to facilitate comparison. Genes located on the positive (+) and negative (−) strands are shown above and below the horizontal line, respectively. Different colors represent protein-coding genes, tRNAs, rRNAs, and the annotated A + T-rich region. The available *C. sareptanus* record lacks the region containing *trnQ* and *trnI*, and no A + T-rich region is annotated in that sequence. Gene blocks are schematic and are not proportional to nucleotide lengths.

Comparative analysis of mitochondrial gene organization among four *Cephus* species revealed a conserved arrangement of the annotated protein-coding and ribosomal RNA genes (Figure 4). In *C. nigrinus*, *C. cinctus*, and *C. pygmeus*, the IQM-associated rearrangement was characterized by the placement of trnQ before *trnI* near *nad2* and the translocation of trnM to the region immediately downstream of rrnS. In *C. sareptanus*, the available sequence does not include the region containing trnQ and *trnI*, preventing direct assessment of this portion of the rearrangement; however, trnM is annotated downstream of rrnS. The available GenBank annotations therefore indicate conservation of the rearranged gene organization among the examined *Cephus* species, with comparisons limited by incomplete terminal sequences.

**Figure 5:**
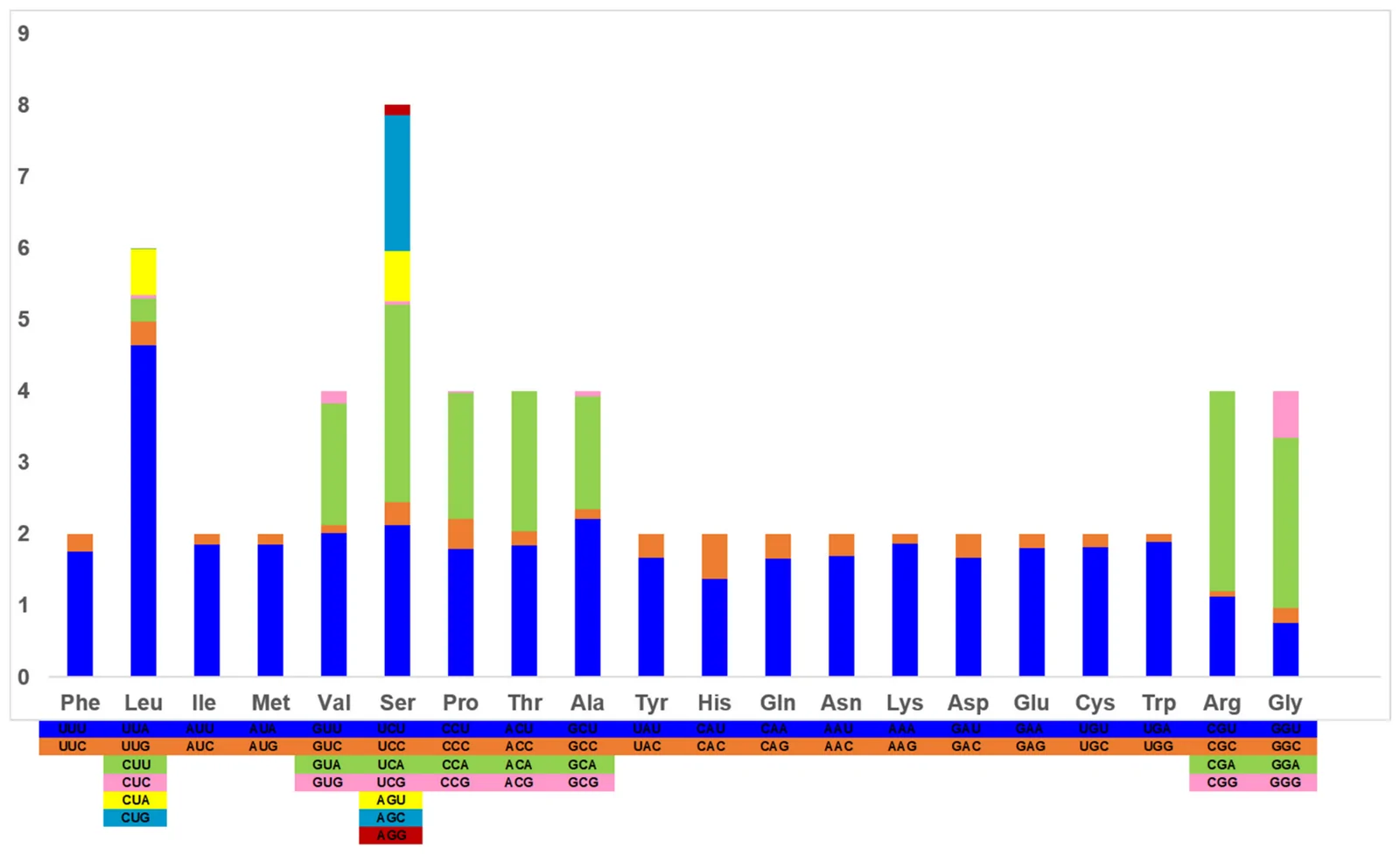
Relative synonymous codon usage (RSCU) in the 13 mitochondrial protein-coding genes of *Cephus nigrinus*. RSCU values represent the relative usage of synonymous codons for each amino acid rather than the absolute abundance of the corresponding amino acids. Individual codons are distinguished by the colors indicated in the figure legend.

The combined length of the 13 protein-coding genes (PCGs) was 11,121 bp. Relative synonymous codon usage (RSCU) analysis revealed a preference for A- and U-rich synonymous codons (Figure 5). UUA (Leu) showed the highest RSCU value (4.64), followed by CGA (Arg; 2.80) and UCA (Ser; 2.76). However, the most abundant codons in terms of absolute counts were AUU (Ile; 415 occurrences) and UUA (Leu; 404 occurrences). These findings distinguish relative synonymous codon preference from absolute codon frequency.

Phylogenetic analyses were performed using a concatenated mitochondrial dataset comprising 13 protein-coding genes, 22 tRNAs, and two rRNAs from 17 taxa (16,619 aligned nucleotide positions). The maximum-likelihood (ML) and Bayesian inference (BI) analyses recovered *C. nigrinus* and *C. pygmeus* as sister taxa. This sister-group relationship received strong support in the ML analysis (ultrafast bootstrap = 100). However, the placement of the *C. nigrinus*–*C. pygmeus* pair relative to *C. cinctus* was less strongly supported (ultrafast bootstrap = 60). Therefore, while the sister relationship between *C. nigrinus* and *C. pygmeus* was consistently recovered, the deeper relationships among the examined *Cephus* species should be interpreted cautiously. The ML and BI phylogenetic trees are presented separately to facilitate comparison of their topologies and support values.

Exploratory analyses of mitochondrial protein-coding genes revealed differences in substitution patterns among codon positions. Third codon positions exhibited a pronounced discrepancy between transitions and transversions across increasing phylogenetic distances, with transitions showing a tendency to plateau or decrease in more divergent sequence comparisons. This pattern was consistent with a greater degree of substitution saturation at third codon positions than at first and second positions. Paired-sequence symmetry analyses also indicated departures from compositional homogeneity in the examined mitochondrial dataset. An additional ML analysis excluding third codon positions was conducted to assess the robustness of the inferred relationships. However, this analysis did not provide a clear resolution of the weakly supported deeper relationships within *Cephus*. These findings suggest that substitutional saturation and compositional heterogeneity should be considered when interpreting mitochondrial phylogenetic relationships. The exploratory substitution saturation plots, paired-sequence symmetry test results, and supplementary ML sensitivity analysis are provided in Supplementary Figure S1, Supplementary Table S1, and Supplementary Figure S2, respectively.

## 4. Discussion

The 16,782-bp mitochondrial sequence of *C. nigrinus* contains the typical complement of 37 mitochondrial genes and an annotated A + T-rich region of 1295 bp. Comparative examination of GenBank records revealed differences in the lengths of the available mitochondrial sequences of *C. cinctus* (19,339 bp; FJ478173), *C. nigrinus* (16,782 bp; BK071385), *C. pygmeus* (16,145 bp; KM377623), and *C. sareptanus* (15,212 bp; KM377624). However, the records of *C. pygmeus* and *C. sareptanus* represent incomplete mitochondrial sequences, and their deposited lengths should not be interpreted as complete mitochondrial genome sizes. The A + T-rich regions annotated in the records of *C. cinctus*, *C. nigrinus*, and *C. pygmeus* are 3873 bp, 1295 bp, and 646 bp long, respectively. Although these annotations indicate variation in the recovered non-coding regions, tandem repeats may be incompletely resolved in assemblies derived from short sequencing reads. Insect mitochondrial genomes are typically organized as circular DNA molecules [2,6], and the gene content and organization of the assembled *C. nigrinus* mitogenome are consistent with the mitochondrial genome architecture reported for related cephid species [17,22]. However, the control region is known to exhibit considerable structural and length variation in Cephidae [17,22], and repetitive sequences may complicate its complete reconstruction from short-read sequencing data. Accordingly, although a 1295-bp A + T-rich region was identified in the present assembly, its complete repeat structure and the precise circularization junction remain to be independently validated.

The mitochondrial gene arrangement of *C. nigrinus* differs from the putative ancestral insect mitochondrial gene order, particularly in the organization of the ancestral trnI–trnQ–trnM (IQM) cluster. In the ancestral arrangement, these three tRNA genes are located near *nad2*, whereas the rearranged configuration observed in *C. nigrinus* consists of trnQ preceding trnI upstream of *nad2*, with trnM relocated to the region immediately downstream of rrnS. This organization is consistent with the IQM-associated rearrangement previously reported in Cephinae [9,17,18,20].

Our comparative analysis of four *Cephus* species further supports conservation of this derived gene organization. The available GenBank annotations of *C. cinctus*, *C. nigrinus*, and *C. pygmeus* exhibit the same relative positions of trnQ, trnI, and trnM. Although the partial mitochondrial sequence of *C. sareptanus* does not include the terminal region containing trnQ and trnI, trnM is also annotated downstream of *rrnS*. These findings agree with previous studies identifying the IQM-associated rearrangement as a conserved feature of Cephinae [20].

The observed gene order is compatible with a historical reorganization involving the relative positions of trnI and trnQ and the translocation of trnM. However, the present comparative data do not establish the precise molecular mechanism or the number of rearrangement events involved. In addition, rearrangements involving the trnT–trnP cluster and tRNA duplications reported in other Cephinae [20] were not identified in the deposited *C. nigrinus* annotation. Further taxonomic sampling and detailed comparisons of mitochondrial gene boundaries will be necessary to clarify the evolutionary history of these structural changes.

An unusual elongation of the mitochondrial small-subunit ribosomal RNA gene (rrnS) has previously been reported in *Cephus* and other members of Cephini [17,18]. In the present study, the annotated rrnS gene of *C. nigrinus* was 987 bp long, consistent with the relatively elongated rrnS sequences described in related taxa. Previous studies identified insertions in domains I and II of rrnS in *C. pygmeus* and *C. sareptanus* [17]. However, the presence, location, and structural correspondence of these insertions in *C. nigrinus* were not specifically investigated. Therefore, the observed rrnS length is compatible with the previously reported pattern, but does not independently establish conservation of the same insertion events.

The mitochondrial sequence of *Cephus nigrinus* exhibited a pronounced A + T bias (81.38%), with the highest A + T content observed at third codon positions (90.15%). Nucleotide composition and strand asymmetry were broadly consistent with patterns reported for other cephid and symphytan mitochondrial genomes [11,17,20]. The predominance of A- and U-rich synonymous codons, particularly UUA (Leu), was also reflected in the relative synonymous codon usage (RSCU) analysis. These patterns may be influenced by nucleotide substitution biases and strand-specific mutational processes, as discussed for insect mitochondrial genomes [2,15]. However, nucleotide composition and synonymous codon usage alone do not provide direct evidence of relaxed purifying selection or adaptive evolution. Accordingly, no conclusions regarding the strength or direction of selection are drawn from these descriptive analyses.

Previous studies have reported parallel amino acid substitutions in mitochondrial protein-coding genes of Cephini [18]. Such substitutions were not specifically tested in *C. nigrinus* in the present study, and their presence or potential adaptive significance cannot be inferred from the available RSCU and nucleotide-composition results. Further comparative protein-sequence analyses would be required to investigate these evolutionary patterns.

**Figure 6:**
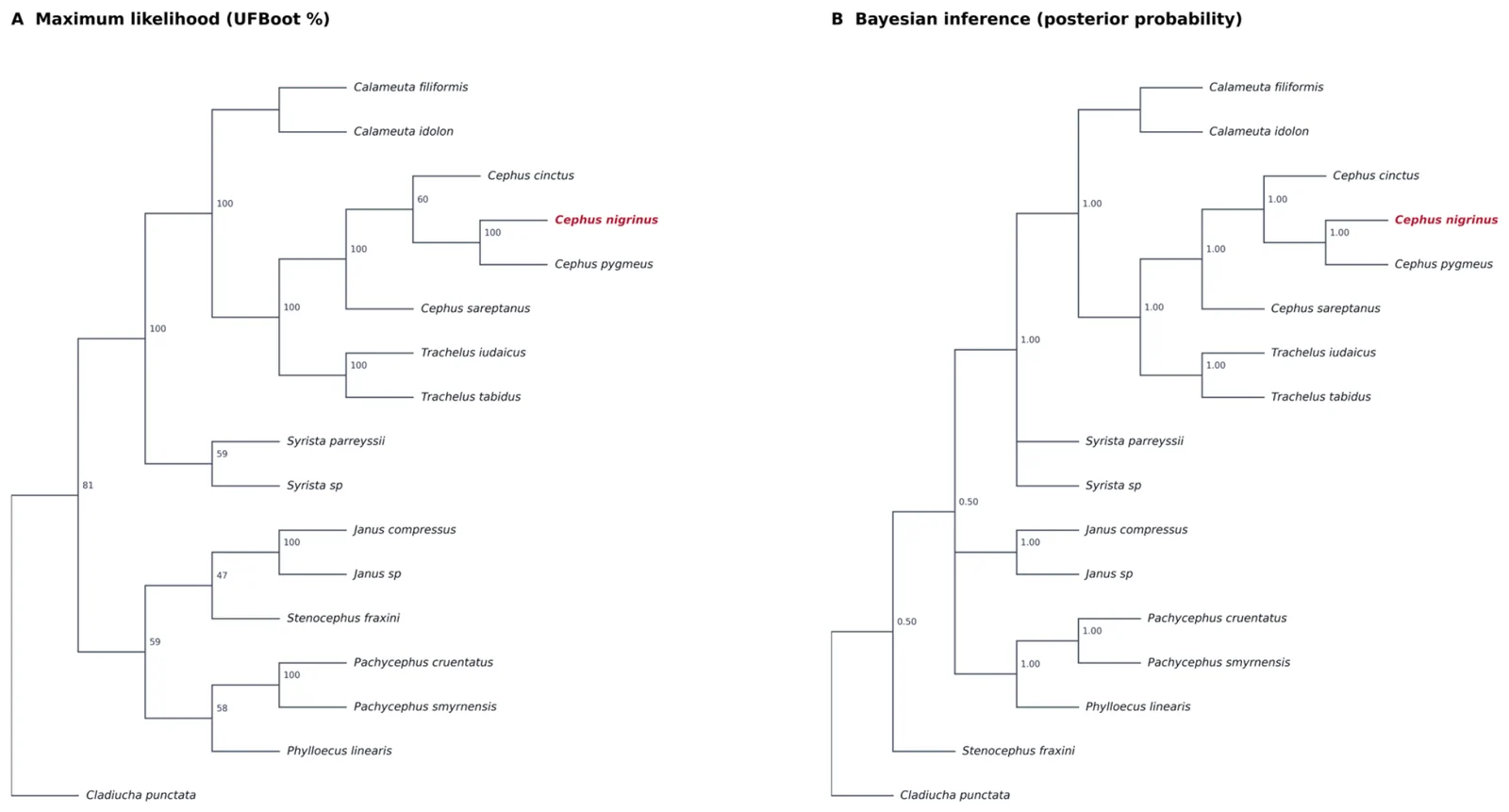
Phylogenetic relationships inferred from the concatenated mitochondrial dataset comprising 13 protein-coding genes, 22 tRNAs, and two rRNAs (16,619 aligned nucleotide positions). (**A**) Maximum-likelihood (ML) tree showing ultrafast bootstrap support percentages. (**B**) Bayesian inference (BI) consensus tree showing posterior probabilities. *Cephus nigrinus* is highlighted in red. Trees are rooted using *Cladiucha punctata* and are displayed as cladograms; branch lengths are not proportional to the estimated number of substitutions.

Phylogenetic analyses of the concatenated mitochondrial dataset recovered *C. nigrinus* and *C. pygmeus* as sister taxa under both maximum likelihood (ML) and Bayesian inference (BI) (Figure 6). This relationship received strong support (ML ultrafast bootstrap = 100; BI posterior probability = 1.00). In contrast, the placement of the *C. nigrinus*–*C. pygmeus* lineage relative to *C. cinctus* received substantially lower support in the ML analysis (ultrafast bootstrap = 60), despite a Bayesian posterior probability of 1.00. This discrepancy indicates that the deeper relationships among the sampled *Cephus* species should be interpreted with caution rather than regarded as unequivocally resolved.

The relatively high nucleotide divergence and pronounced A + T bias of mitochondrial sequences may complicate phylogenetic reconstruction, particularly at rapidly evolving synonymous sites. Exploratory analyses of the individual PCG codon positions revealed a pattern consistent with greater substitution saturation at third codon positions than at first and second positions. Compositional heterogeneity was also detected in the examined dataset. Although these findings suggest that rapidly evolving and compositionally heterogeneous sites may influence phylogenetic resolution, they do not establish a specific cause for the weakly supported node.

The additional sensitivity analysis using the first and second PCG codon positions did not resolve the uncertainty associated with the deeper *Cephus* relationships. Accordingly, the consistent recovery of the *C. nigrinus*–*C. pygmeus* sister-group relationship is considered more reliable than the precise branching order of the remaining congeners. Further analyses using expanded taxon sampling, additional independent molecular markers, and models that better accommodate compositional heterogeneity would be valuable for resolving these relationships. Although previous studies have discussed relationships between mitochondrial phylogeny and host-plant associations within Cephini [20,23], the present results do not provide sufficient evidence to infer a host-associated evolutionary split.

The mitochondrial gene organization of *C. nigrinus* is consistent with previous comparative studies showing that tRNA rearrangements represent conserved structural characters within Cephinae [17,20]. In particular, the IQM-associated rearrangement appears to be shared among several examined cephine lineages, whereas the overall arrangement of protein-coding and ribosomal RNA genes remains comparatively stable. However, the phylogenetic distribution of these rearrangements cannot be fully established from the currently available mitochondrial genomes. Expanded taxonomic sampling, particularly of Hartigiini, Athetocephinae, and Australcephinae, will be important for testing the broader evolutionary distribution of these structural characters [20].

This species has also been documented in regional faunistic surveys [19], providing additional context for interpreting its phylogenetic placement within Cephidae. The comparative analysis supports conservation of the IQM-associated gene arrangement among the examined *Cephus* species, whereas the available annotations suggest variation in non-coding regions. Nevertheless, the extent of control-region variation remains uncertain because repetitive sequences may not be fully resolved by short-read assemblies. Further mitogenomic sampling, including additional representatives of Cephini and other cephine lineages, will be necessary to assess the evolutionary distribution of these structural features. The reconstruction of the *C. nigrinus* mitochondrial sequence from publicly available whole-genome reads also demonstrates the value of existing genomic datasets for expanding comparative taxonomic resources.

## 5. Conclusions

The mitochondrial genome assembly of *C. nigrinus*, reconstructed from publicly available Illumina sequencing data, provides an additional resource for comparative mitogenomic studies of Cephidae. The 16,782-bp sequence contains the typical complement of 37 mitochondrial genes and exhibits the IQM-associated gene rearrangement previously reported in Cephinae. Comparative analysis of four *Cephus* species supports conservation of the derived arrangement, particularly the placement of trnQ before trnI near *nad2* and the relocation of trnM downstream of rrnS.

Maximum-likelihood and Bayesian phylogenetic analyses consistently recovered *C. nigrinus* and *C. pygmeus* as sister taxa. However, deeper relationships within *Cephus* received unequal support between the two approaches and should be interpreted cautiously. The annotated A + T-rich region of *C. nigrinus* spans 1295 bp, although its complete repeat structure and the circularization junction remain to be independently validated. Overall, these findings expand the available mitochondrial genomic resources for Cephinae and highlight the importance of additional taxonomic sampling and independent assembly validation for understanding mitochondrial genome evolution within Cephidae.

## Figures and Tables

**Table 1:** Taxa included in the phylogenetic analyses and their GenBank accession numbers. The following GenBank accessions were used: BK071385 (this study), KX907844 [20], OL757402 (GenBank direct submission), ON153182 (GenBank direct submission), KX907845 [20], NC_065664 (GenBank direct submission), KX907843 [20], OK104785 [47], OL889929 (GenBank direct submission), FJ478173 [21], KM377623 [17], KM377624 [17], KX257357 [18], NC_032072 [18], KT260167 [22], NC028446 [22], and NC_062838 [48].

	Superfamily	Family	Species	Genus	Accession Number
Ingroup	Cephoidea	Cephidae	*Janus compressus*	*Janus*	KX907844
			*Janus* sp.		OL757402
			*Stenocephus fraxini*	*Stenocephus*	ON153182
			*Pachycephus cruentatus*	*Pachycephus*	KX907845
			*Pachycephus smyrnensis*		NC_065664
			*Phylloecus linearis*	*Phylloecus*	KX907843
			*Syrista parreyssii*	*Syrista*	OK104785
			*Syrista* sp.		OL889929
			*Cephus cinctus*	*Cephus*	FJ478173
			*Cephus nigrinus*		BK071385 (this study)
			*Cephus pygmeus*		KM377623
			*Cephus sareptanus*		KM377624
			*Trachelus iudaicus*	*Trachelus*	KX257357
			*Trachelus tabitus*		NC_032072
			*Calameuta filiformis*	*Calameuta*	KT260167
			*Calameuta idolon*		NC_028446
Outgroup	Tenthredinidae	Allantinae	*Cladiucha punctata*	*Cladiucha*	NC_062838

**Table 2:** Mitogenome organisations of *Cephus nigrinus.* + and − refer to heavy and light strands, respectively; IGN refers to intergenic nucleotides. Minus indicates overlapping sequences between adjacent genes.

Gene/Region	Strand	Start	End	Length (bp)	Start Codon	Stop Codon	IGN (bp)	Anticodon
trnQ	−	1	69	69	—	—	0	AAC
trnI	+	139	206	68	—	—	69	CUA
*nad2*	+	322	1320	999	ATT	TAA	115	
trnW	+	1320	1387	68	—	—	−1	AGU
trnC	−	1380	1450	71	—	—	−8	CGU
trnY	−	1469	1535	67	—	—	18	CAU
*cox1*	+	1574	3112	1539	ATG	TAA	38	
trnL2	+	3108	3175	68	—	—	−5	AUU
*cox2*	+	3176	3856	681	ATG	TAA	0	
trnK	+	3877	3948	72	—	—	20	GAA
trnD	+	3961	4029	69	—	—	12	CAG
*atp8*	+	4039	4203	165	ATA	TAA	9	
*atp6*	+	4197	4871	675	ATG	TAA	−7	
*cox3*	+	4891	5679	789	ATG	TAA	19	
trnG	+	5703	5775	73	—	—	23	AGG
*nad3*	+	5776	6129	354	ATT	TAA	0	
trnA	+	6130	6194	65	—	—	0	ACG
trnR	+	6202	6264	63	—	—	7	UCG
trnN	+	6262	6331	70	—	—	−3	GUU
trnS1	+	6332	6402	71	—	—	0	AUC
trnE	+	6454	6518	65	—	—	51	AAG
trnF	−	6481	6549	69	—	—	−38	CUU
*nad5*	−	6578	8260	1683	ATT	TAG	28	
trnH	−	8264	8329	66	—	—	3	CAC
*nad4*	−	8334	9689	1356	ATG	TAA	4	
*nad4l*	−	9683	9976	294	ATG	TAA	−7	
trnT	+	9996	10,069	74	—	—	19	ACA
trnP	−	10,075	10,139	65	—	—	5	ACC
*nad6*	+	10,232	10,732	501	ATT	TAA	92	
*cob*	+	10,732	11,874	1143	ATG	TAA	−1	
trnS2	+	11,875	11,942	68	—	—	0	CGA
*nad1*	−	11,963	12,904	942	ATA	TAG	20	
trnL1	−	12,911	12,978	68	—	—	6	AUC
rrnL	−	12,979	14,320	1342	—	—	0	
trnV	−	14,353	14,418	66	—	—	32	AUG
rrnS	−	14,432	15,418	987	—	—	13	
trnM	+	15,419	15,487	69	—	—	0	GUA
A + T-rich region	+	15,488	16,782	1295	—	—	0	

**Table 3:** Nucleotide composition of the mitogenomes of *Cephus nigrinus*.

	T%	C%	A%	G%	A + T%	G + C%	AT-Skew	GC-Skew
Whole mitogenome	38.21	11.80	43.17	6.81	81.38	18.62	0.061	−0.268
Protein coding genes	43.43	10.93	35.10	10.54	78.53	21.47	−0.106	−0.018
First codon position	36.09	10.15	38.20	15.57	74.28	25.72	0.028	0.211
Second codon position	49.35	16.32	21.82	12.51	71.17	28.83	−0.387	−0.132
Third codon position	44.86	6.31	45.29	3.55	90.15	9.85	0.005	−0.280
tRNA genes	41.69	6.68	42.55	9.07	84.25	15.75	0.010	0.151
rRNA genes	45.54	5.19	38.82	10.46	84.35	15.65	−0.080	0.337

## Data Availability

The publicly available Illumina sequencing reads analyzed in this study are deposited in the NCBI Sequence Read Archive under accession SRR23907852 (BioProject PRJNA809169; BioSample SAMN28561985). The mitochondrial genome assembly and annotation generated in this study are available in GenBank under accession BK071385.

## Supplementary Materials

The following supporting information can be downloaded at: https://www.mdpi.com/article/10.3390/biology15212009/s1, Figure S1. Exploratory substitution saturation analysis of mitochondrial protein-coding genes based on transitions and transversions at the first, second, and third codon positions. Figure S2. Maximum-likelihood phylogenetic tree reconstructed using the first and second codon positions of mitochondrial protein-coding genes. Table S1. Results of paired-sequence symmetry tests assessing nucleotide-composition heterogeneity and substitutional symmetry in the mitochondrial dataset. Table S2. Partitioning scheme and nucleotide substitution models selected by PartitionFinder for the concatenated mitochondrial dataset.
